# Cryptococcosis in a patient with HIV infection

**DOI:** 10.1186/1471-2334-13-S1-P109

**Published:** 2013-12-16

**Authors:** Vasile Benea, Simona Roxana Georgescu, Mircea Tampa, Mihaela-Anca Benea-Mălin, Diana Leahu, Cristina Răileanu, Șerban Benea

**Affiliations:** 1 Department of Dermatology, Clinical Hospital of Infectious and Tropical Diseases “Dr. Victor Babeş”, Bucharest, Romania; 2 National Institute for Infectious Diseases “Prof. Dr. Matei Balş”, Bucharest, Romania

## Background

*Cryptococcus neoformans* is encapsulated yeast with worldwide distribution, which may cause a self-limited pulmonary infection or disseminate (especially to the meninges, but sometimes to the skin, bones, viscera, or other sites). The infection is acquired by inhalation of contaminated soil.

Cryptococcosis is a defining opportunistic infection for AIDS; also at increased risk for infection are patients with Hodgkin’s or other lymphomas, sarcoidosis, or those receiving long-term corticosteroid therapy. Progressive disseminated cryptococcosis also sometimes affects men over 40 years that aren’t obviously immunocompromised.

Most cryptococcal infections have a self-limited, subacute or chronic course; in AIDS patients cryptococcal infection may present with acute, severe pneumonia. The brain is the most common organ infected by the hematogenous route; cryptococcal meningitis is an important complication of AIDS. Disseminated cutaneous involvement occurs also by hematogenous dissemination; it causes pustular, papular, nodular, or ulcerated lesions, sometimes resembling acne, molluscum contagiosum, or basal cell carcinoma. The initial treatment is based on amphotericin B and flucytosine; oral fluconazole is needed for chronic suppressive therapy, especially in AIDS patients.

## Case report

We present a 32 years old man who was referred for a disseminated polymorph eruption with papular, nodular and ulcerated lesions. The serology for HIV infection was positive and the biopsy identified encapsulated yeasts. Because the patient had fever, headache, confusion, agitation and blurred vision, he was transferred in a neurology clinic.

